# Inositol(s) from Bench to Bedside in Endocrinology and Gynecology

**DOI:** 10.1155/2017/8515703

**Published:** 2017-04-04

**Authors:** Vittorio Unfer, John E. Nestler, Zdravko A. Kamenov, Nikos Prapas, Fabio Facchinetti

**Affiliations:** ^1^Department of Medical Sciences, IPUS-Institute of Higher Education, Chiasso, Switzerland; ^2^Department of Medicine and Department of Obstetrics and Gynecology, Virginia Commonwealth University, Richmond, VA, USA; ^3^Clinic of Endocrinology, Alexandrovska University Hospital, Medical University, Sofia, Bulgaria; ^4^IAKENTRO, Infertility Treatment Center, Thessaloniki, Greece; ^5^Mother-Infant Department, University of Modena and Reggio Emilia, Modena, Italy

This special issue is aimed at providing innovative scientific data and discussing in-depth results and findings so far available to further clarify some pivotal scientific topics in the field of inositol(s). The authors focused their attention on three essential molecules belonging to this primordial group of ubiquitary chemical compounds: myoinositol and D-chiroinositol, two stereoisomeric forms of inositol(s), and inositol hexakisphosphate, a metabolite of myoinositol. These molecules are involved in many physiological functions and can be used for numerous therapeutic aims, in some cases exploiting their feature to exist in different enantiomeric forms, each one with specific tasks. Plenty of experimental and clinical data have shown that myoinositol alone, or (depending on the therapeutic aim) in association with D-chiroinositol in a physiologic ratio (40 : 1) between the two molecules, plays a pivotal role in treating several pathologies such as PCOS, metabolic syndrome, gestational diabetes, thyroid disorders, and infertility. Furthermore, myoinositol allows also improving substantially the outcomes of assisted reproduction technology (ART). Finally, myoinositol and inositol hexakisphosphate have demonstrated very promising anticancer activities as shown by numerous studies.

Although an increasing number of researches and investigations were realized on inositol(s) physiological properties as well as on the pathological conditions caused by their imbalance, a few points had to be reaffirmed and examined in-depth. The effort by the authors to address these issues was stimulated by the necessity to clear up a couple of improper ideas about the physiological functions carried out by myoinositol and D-chiroinositol. As a matter of fact, we may say that so far, not enough attention has been paid to this issue, at least in some cases. Despite the fact that it was well shown that such molecules play basic but very different roles in regulating various metabolic pathways and hormonal signaling, a few proposals of therapeutic application reveal to be still not appropriately focused. It is essential to highlight that myoinositol, the most represented and important stereoisomeric form of inositol, stands out for its key roles as leading molecule in some physiological areas, whereas different integrative functions are carried out by D-chiroinositol, synthetized from myoinositol under the enzymatic control of an epimerase. To get straight to the point, whereas the activation of glucose transporters and glucose utilization take place under the regulation of myoinositol, glycogen synthesis is mainly controlled by D-chiroinositol. On the other hand, in the ovary, myoinositol regulates glucose uptake and FSH signaling, while D-chiroinositol modulates insulin-induced androgen synthesis [1, 2]. In physiological conditions, the intracellular pool of inositol(s) in human ovaries and testis contains 99% of myoinositol whereas the remaining part is D-chiroinositol [2, 3]. It means that myoinositol is essential qualitatively and quantitatively for the functioning of the reproductive system.

It was discovered that an imbalance between myoinositol and D-chiroinositol concentrations occurs in the ovary of PCOS women, with a myoinositol deficiency, which might impair the FSH signaling [2, 3]. In these conditions, glucose uptake and metabolism in oocytes and follicular cells are negatively affected, thereby compromising oocyte quality that depends on the availability of adequate amounts of myoinositol [2, 3]. The improvement of ovarian function, as well as hormonal and metabolic parameters, was demonstrated after myoinositol treatment in PCOS women [2, 3]. On the other hand, high doses of D-chiroinositol alone, administered to PCOS subjects, were found significantly detrimental for oocytes and therefore for fertility [2, 3]. Finally, it is not to be forgotten that myoinositol is a well-established safe molecule [4]. By now, it is possible to outline a comprehensive framework and bring about the best treatment for PCOS in keeping with the above cited findings, specifying some milestones useful for the therapeutic application. Many articles of the special issue contributed to further shed light on this topic and strengthen several points with original studies and insightful reviews of up-to-date scientific literature. In our systematic review (V. Unfer et al.), we analysed recent randomized clinical trials of inositol(s) in PCOS, in particular myo- and D-chiroinositol, aiming at better elucidating their physiological involvement in PCOS and potential therapeutic use, alone and in conjunction with assisted reproductive technologies, in the clinical treatment of women with PCOS. Other articles reported the results of a study on the putative role due to D-chiroinositol and its messenger in insulin resistance in women with PCOS independent of obesity (K. I. Cheang et al.) and an in-depth investigation on the possible involvement of inositol messengers in pre-eclampsia (S. Kunjara et al.). Other contributions were focused on PCOS therapy (and in a lesser extent on gestational diabetes and metabolic syndrome therapy), regarding the improvement of the metabolic profile and also the restoration of patients' fertility (A. S. Laganà et al., G. Muscogiuri et al.). On the same topic besides the cited reviews, various clinical studies are presented and carried out in adults (E. Benelli et al., A. C. Ozay et al., and S. Salehpour et al.) and teenagers (L. Pkhaladze et al.). Myoinositol was proven to be efficacious in increasing significantly the overall rate of live births in female mice (N. Kuşcu et al.), and in treating infertility in men, with or without metabolic syndrome (two studies by M. Montanino et al., M. Palmieri et al.), and in women affected or not by PCOS (D. Garg et al., B. Lesoine et al., P.-A. Regidor and A. E. Schindler, G. Simi et al., S. G. Vitale et al., and A. Wdoviak). Myoinositol, in association with folic acid, administered during pregnancy, was found to be very efficacious in warding off the occurrence of neural tube defects in infants (P. Cavalli and E. Ronda). Furthermore, other studies and reviews investigated the promising effects of myoinositol and selenium in the treatment of autoimmune thyroiditis (M. Nordio and S. Basciani), myoinositol plus D-chiroinositol, at the physiological ratio (40 : 1), for the therapy of type 2 diabetes (B. Pintaudi et al.) and for improving insulin resistance in obese male children (M. Mancini et al.). Also, myoinositol and its metabolite, inositol hexakisphosphate, deserve to be carefully taken into consideration. Both these molecules exert a wide range of critical activities in physiological and pathological settings. Deregulated inositol(s) metabolism was observed in a number of diseases, including cancer, where they modulate different critical pathways. Inositol hexakisphosphate was found to inhibit growth and invasiveness of several cancer types, whereas both inositol hexakisphosphate and myoinositol exert substantial chemopreventive effects in vitro and in vivo. Of note, myoinositol is able to significantly synergize with inositol hexakisphosphate in inducing cancer inhibition. Moreover, they are provided with antioxidant activity, moderate in myoinositol, and very high in inositol hexakisphosphate. The second one acts as a chelator of harmful trace elements, such as iron, uranium, nickel, copper, and other potentially toxic elements, often involved in tumor onset. In addition to that, these inositols modulate processes such as mRNA transcription, chromatin remodeling, cytoskeleton configuration, and p53 activity, to name a few. Thereby, inositol hexakisphosphate or myoinositol alone, or in synergy, can be excellent therapeutic agents that protect from cancer and other threats to human health (M. Bizzarri et al., R. Lauretta et al.).

We outlined the framework in which our special issue is placed, providing a guiding thread for reading the various contributions according to the topics covered by the authors. These articles convey a clear picture and can elicit new insights in researchers and readers concerning the studies on inositol(s).

*Vittorio Unfer* *John E. Nestler* *Zdravko A. Kamenov* *Nikos Prapas* *Fabio Facchinetti*
