# Corrigendum to “Inositol(s) from Bench to Bedside in Endocrinology and Gynecology”

**DOI:** 10.1155/2017/6451082

**Published:** 2017-12-28

**Authors:** Vittorio Unfer, John E. Nestler, Zdravko A. Kamenov, Nikos Prapas, Fabio Facchinetti

**Affiliations:** ^1^Department of Medical Sciences, IPUS-Institute of Higher Education, Chiasso, Switzerland; ^2^Department of Medicine and Department of Obstetrics and Gynecology, Virginia Commonwealth University, Richmond, VA, USA; ^3^Clinic of Endocrinology, Alexandrovska University Hospital, Medical University, Sofia, Bulgaria; ^4^IAKENTRO, Infertility Treatment Center, Thessaloniki, Greece; ^5^Mother-Infant Department, University of Modena and Reggio Emilia, Modena, Italy

In the Editorial titled “Inositol(s) from Bench to Bedside in Endocrinology and Gynecology” [1], a “Conflicts of Interest” section should have been added as follows:

Conflicts of Interest

Vittorio Unfer, the lead guest editor of the special issue, is an employee of LO.LI. PHARMA SRL. This company markets products containing inositols. John E. Nestler has received travel expenses from LO.LI. PHARMA and SUN Pharmaceuticals. Zdravko A. Kamenov has received travel expenses from LO.LI PHARMA. Fabio Facchinetti has received travel expenses from LO.LI. PHARMA, SUN Pharmaceuticals, and Alkem Laboratories. Nikos Prapas has no interests to declare.
